# Supplementary material for: Reversed thermo-switchable molecular sieving membranes composed of two-dimensional metal-organic nanosheets for gas separation
Source: Nat Commun. 2017 Feb 16;8:14460. doi: 10.1038/ncomms14460 (PMC5316892; doi:10.1038/ncomms14460)
Supplement: Supplementary Information — Supplementary Figures, Supplementary Tables, Supplementary Notes and Supplementary References [file ncomms14460-s1.pdf]

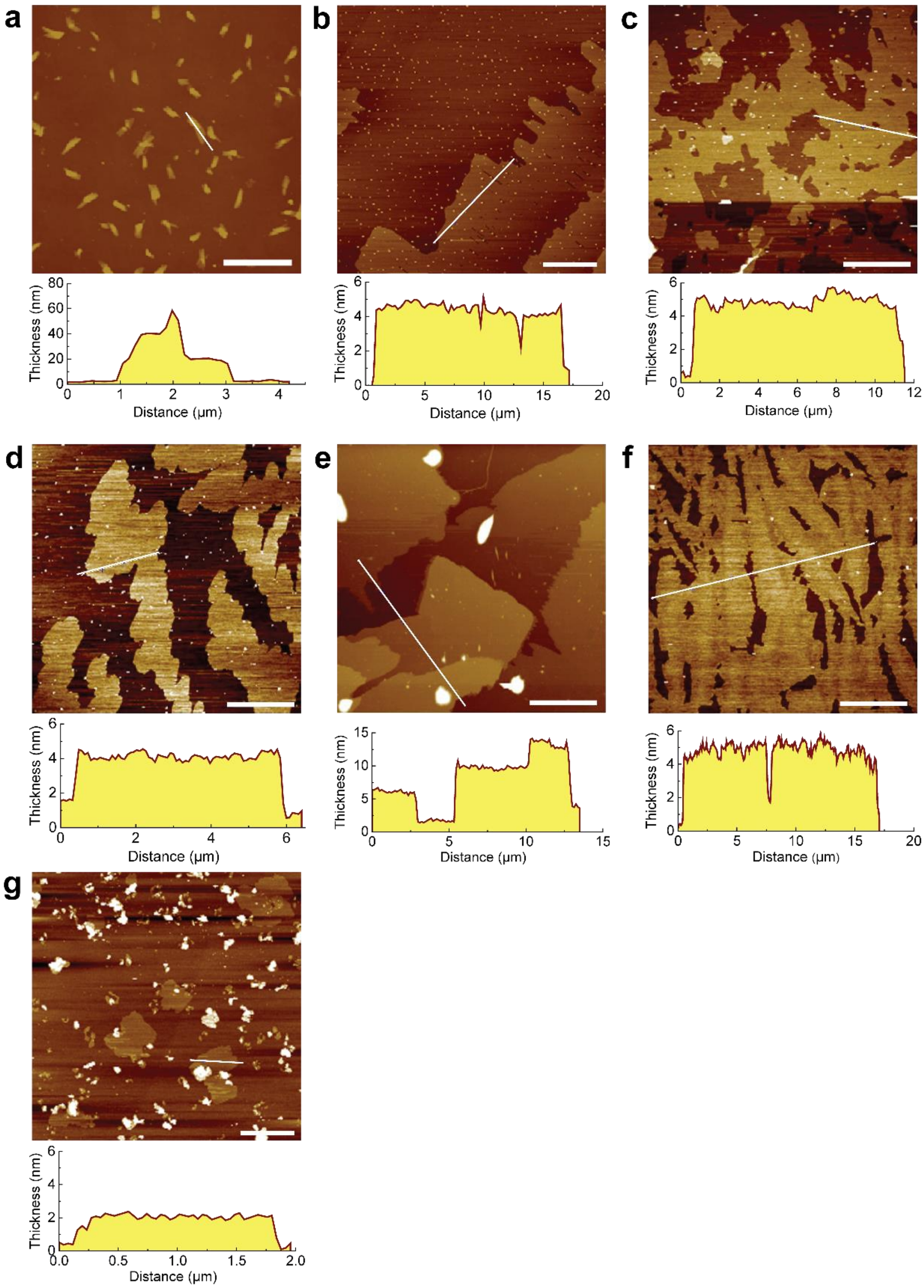

Supplementary Figure 1 | AFM images of exfoliated MAMS-1 nanosheets with different solvents. a, 20-cycle freeze-thaw in water. **b**, 20-cycle freeze-thaw in 20 v% ethanol/water. **c**, 20-cycle freeze-thaw in 80 v% ethanol/water. **d**, 20-cycle freeze-thaw in ethanol. **e**, 10-cycle freeze-thaw in ethanol. **f**, 10-cycle freeze-thaw in hexane. **g**, 30 min sonication in hexane. Scale bars: (a-f), 5 μm; (g), 2 μm.

Supplementary Note 1: No exfoliated MAMS-1 nanosheet can be observed after 20-cycle

freeze-thaw of the dispersed MAMS-1 crystals in water (Supplementary Figure 1a). This result is different from the previous reports regarding the exfoliation of GO nanosheets by the freeze-thaw method, wherein 2-3 layered GO nanosheets can be obtained after 6 freeze-thaw cycles of the dispersed GO powder in water[1, 2]. A possible reason is that water molecules cannot penetrate through the hydrophobic PW1 aperture (*ca.* 0.29 nm) of MAMS-1 into its interlayer region. This causes insufficient water content in the interlayer region and a weak shear force originated from water solidification during freeze-thaw cycles. Sansom and co-workers have demonstrated that water molecules fail to penetrate into the hydrophobic pores if the diameter is less than *ca.* 0.90 nm[3]. Another possible reason is that water (surface tension: 72.8 mN m^{-1}), as a polar solvent, cannot stabilize the exfoliated MAMS-1 nanosheets because of their highly hydrophobic surface caused by the covering nonpolar *tert*-butyl groups. Inversely, it should be much easier for organic solvents with smaller surface tension to wet the hydrophobic interlayer region of MAMS-1 crystals during exfoliation and stabilize the exfoliated MAMS-1 nanosheets. For example, large nanosheets (mainly bilayered with thickness of *ca.* 4 nm) can be easily observed after 20 freeze-thaw cycles in 20 v% ethanol/water mixture (surface tension: 42.3 mN m^{-1}, Supplementary Figure 1b), 80 v% ethanol/water mixture (surface tension: 24.32 mN m^{-1}, Supplementary Figure 1c), and pure ethanol (surface tension: 22.31 mN m^{-1}, Supplementary Figure 1d-e). Considering the hydrophobic lateral plane of MAMS-1 nanosheets, we speculate that organic solvents with small surface tension are beneficial for the exfoliation and stabilization of MAMS-1 nanosheets. This is confirmed by the facile exfoliation of MAMS-1 in hexane by only 10 freeze-thaw cycles (surface tension: 17.94 mN m^{-1}, Supplementary Figure 1f). Sonication of MAMS-1 crystals in hexane suspension can generate a few monolayered MAMS-1 nanosheets (thickness of *ca.* 2 nm), but at the cost of fragmented nanosheets and irregular debris unsuitable for membrane fabrication (Supplementary Figure 1g). It should be noted that many nano-sized fragments or particles still remain in the supernatant even after centrifugation at 10,000 rpm for 20 min, which can be fatal to the fabrication of high-quality 2D MAMS-1 membranes[4]. Therefore, purification of MAMS-1 nanosheets to remove small fragments or particles from large nanosheets becomes necessary.

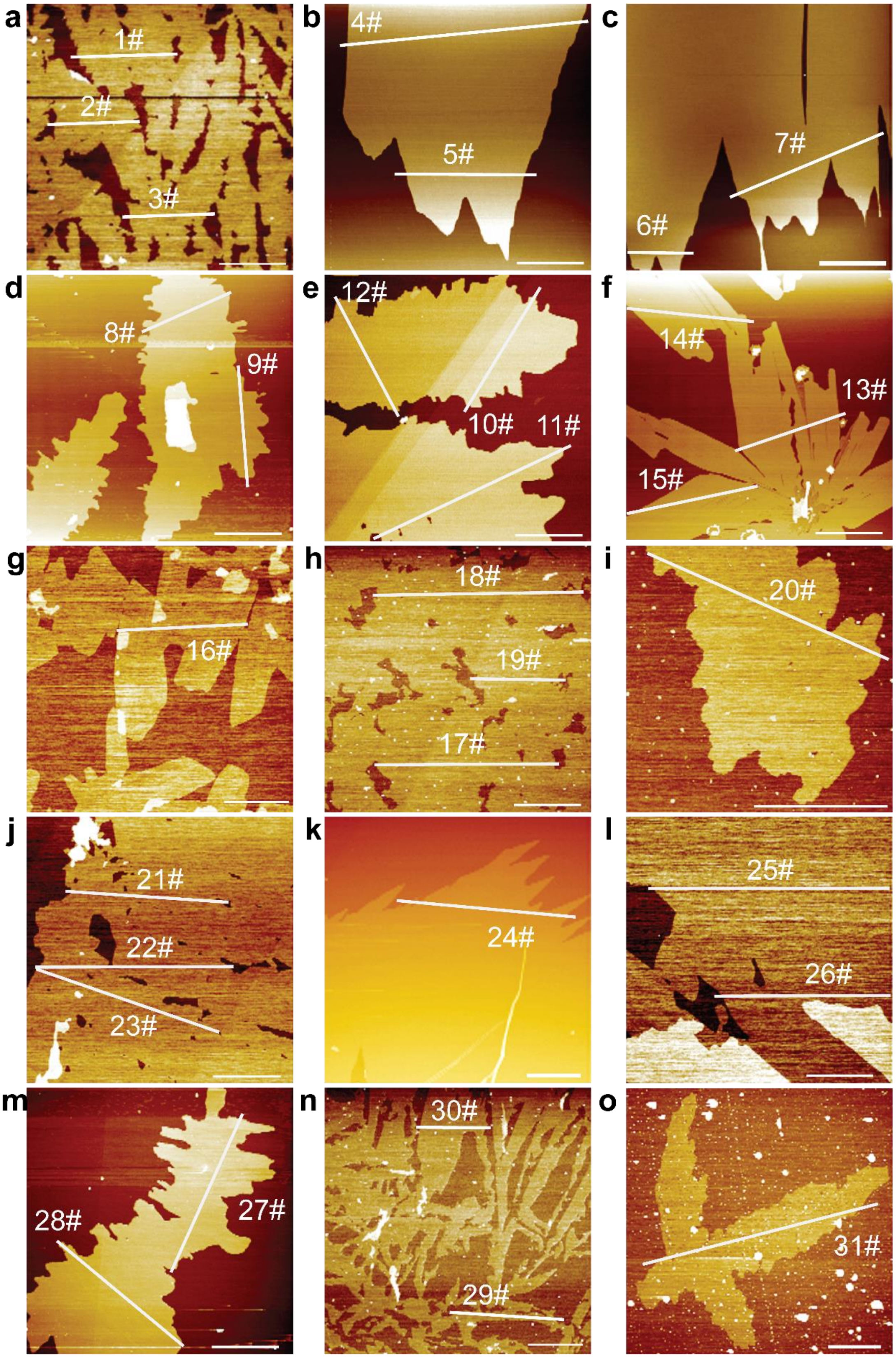
a
1#
2#
3#
b
4#
5#
c
7#
6#
d
8#
9#
e
12#
10#
11#
f
14#
13#
15#
g
16#
h
18#
19#
17#
i
20#
j
21#
22#
23#
k
24#
l
25#
26#
m
27#
28#
n
30#
29#
o
31#

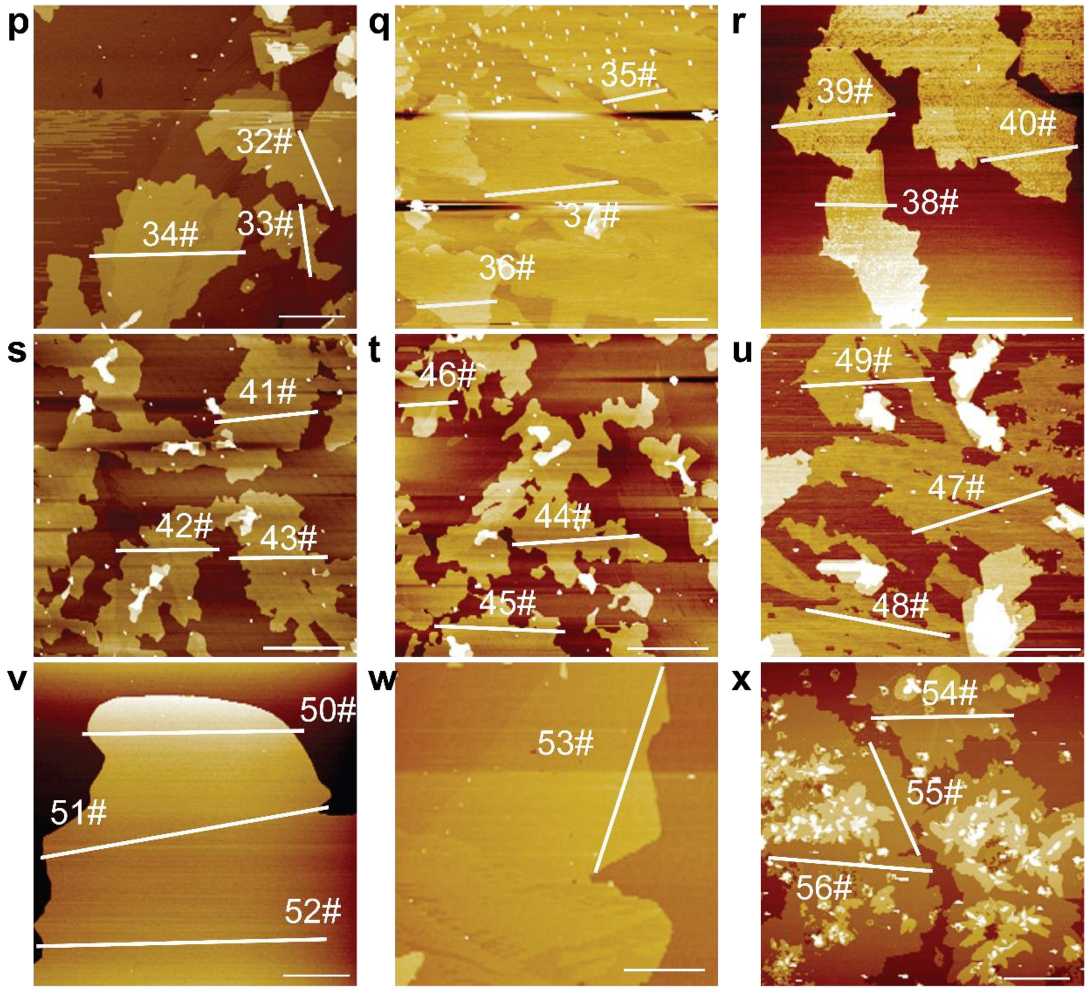

Supplementary Figure 2 | Statistical analysis of the AFM images of exfoliated MAMS-1 nanosheets for thickness and lateral size shown in Figure 2b. Supplementary Figure 2c is also shown as Figure 2e in the main text. Scale bars: (a, b, d-x), 5 μm; (c), 10 μm.

Supplementary Note 2: The thickness and lateral size of exfoliated MAMS-1 nanosheets are summarized from the 56 AFM sites shown above. More than 95 % of them are bilayered with a thickness of *ca.* 4 nm. The lateral size of the exfoliated MAMS-1 nanosheets can be as large as 20 μm shown in Supplementary Figure 2a-c.

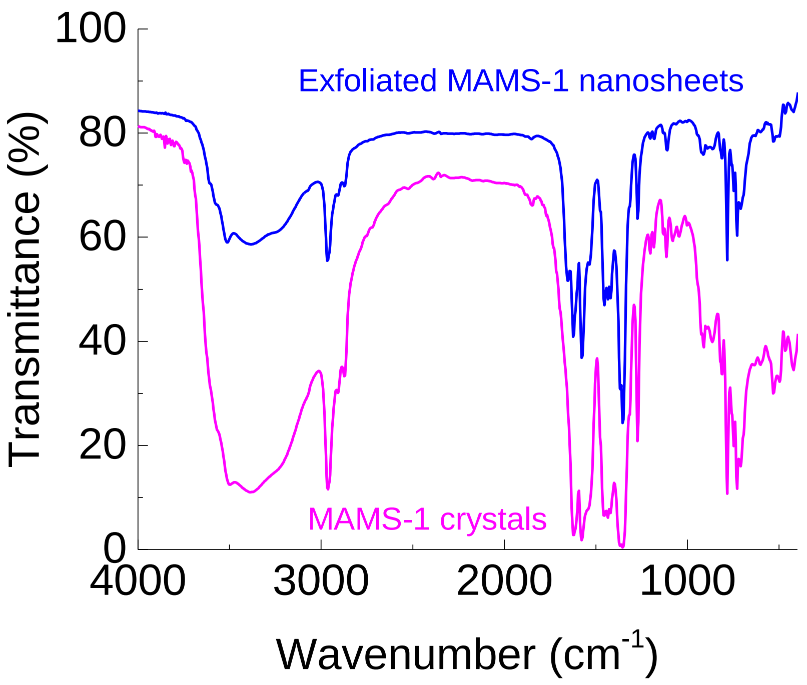

Supplementary Figure 3│FTIR spectra of MAMS-1 crystals and exfoliated MAMS-1 nanosheets.

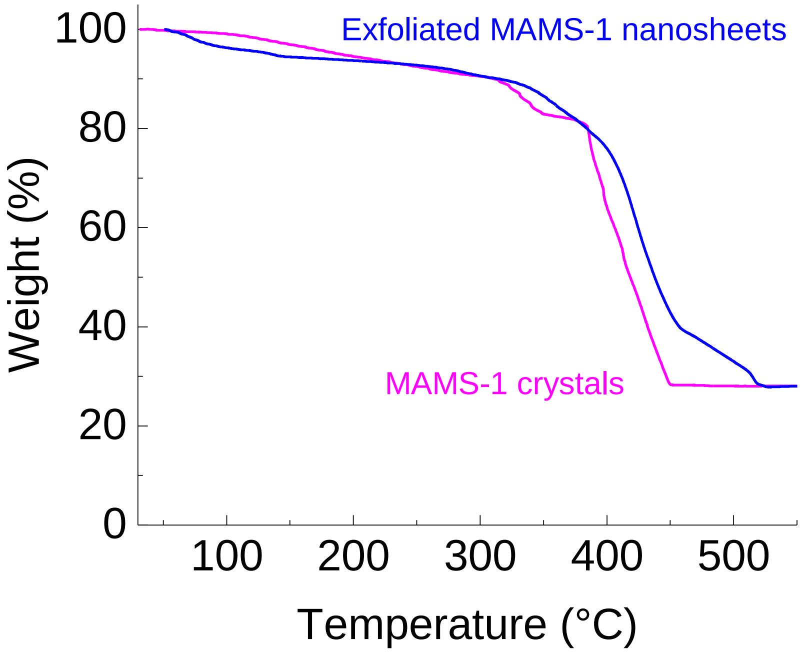

Supplementary Figure 4│TGA curves of MAMS-1 crystals and exfoliated MAMS-1 nanosheets.

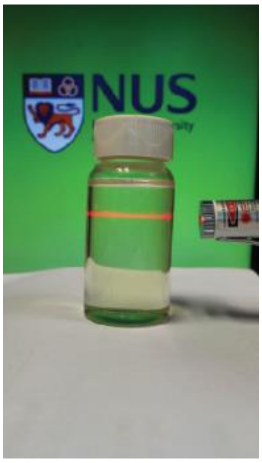

Supplementary Figure 5 | Tyndall effect of MAMS-1 nanosheets suspended in hexane.

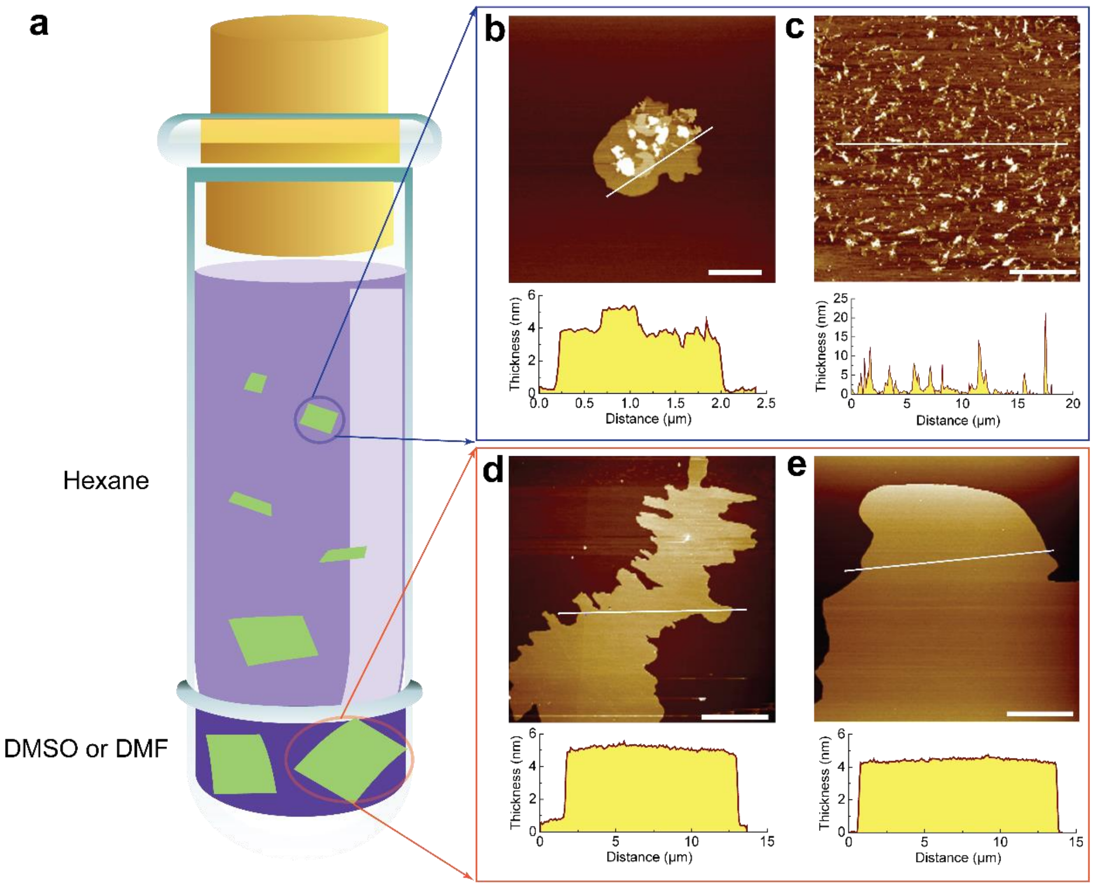

Supplementary Figure 6 | Solvent-selective sedimentation approach for the size-fractionation of MAMS-1 nanosheets. **a**, Illustration for the solvent-selective sedimentation approach. **b**, AFM image of MAMS-1 nanosheets collected from top hexane layer after standing for two weeks. **c**, AFM image of MAMS-1 nanosheets collected from top hexane layer after standing for 4 months. **d**, AFM image of MAMS-1 nanosheets collected from bottom DMF layer of hexane/DMF system after standing for two weeks. This image is also shown as Supplementary Figure 2m. **e**, AFM image of MAMS-1 nanosheets collected from bottom DMSO layer of hexane/DMSO system after standing for 4 months. This image is also shown as Supplementary Figure 2v. Scale bars: (b), 1 μm; (c-e), 5 μm.

Supplementary Note 3: Compared with the large nanosheets collected from bottom DMF or DMSO layer (Supplementary Figure 6d and Supplementary Figure 6e), the nanosheets collected from top hexane layer have much smaller lateral size mixed with nano-sized fragments or particles (Supplementary Figure 6b and Supplementary Figure 6c), confirming the effectiveness of solvent-selective sedimentation approach in the size-fractionation of MAMS-1 nanosheets. Meanwhile, the purified MAMS-1 nanosheets demonstrate excellent dispersion stability in DMSO suspensions without agglomeration or decomposition even for longer than 4 months (Supplementary Figure 6e). The excellent dispersion stability might be due to the intrinsic thermal vibration of MAMS-1 nanosheets, which is identical to that of the freely suspended graphene nanosheets[5, 6].

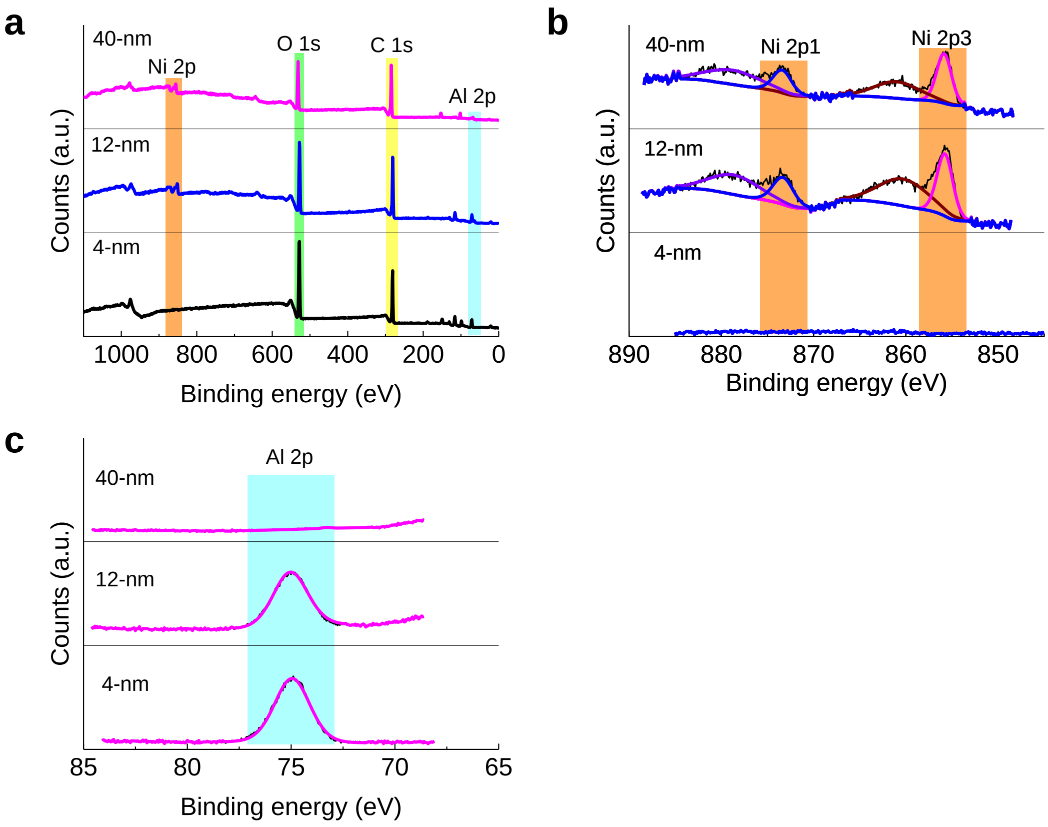

Supplementary Figure 7 | XPS spectra of 4-nm, 12-nm, and 40-nm 2D MAMS-1 membranes fabricated using AAO as the substrates. **a**, Full scan. **b**, Ni 2*p*. **c**, Al 2*p*.

Supplementary Note 4: The presence of Ni element indicates the successful deposition of MAMS-1 nanosheets as the membrane layer, while the presence of Al element confirms the nanometer-thickness of the 12-nm membrane because Al comes from the underlying AAO substrate and XPS has a detection depth of only several nanometers.

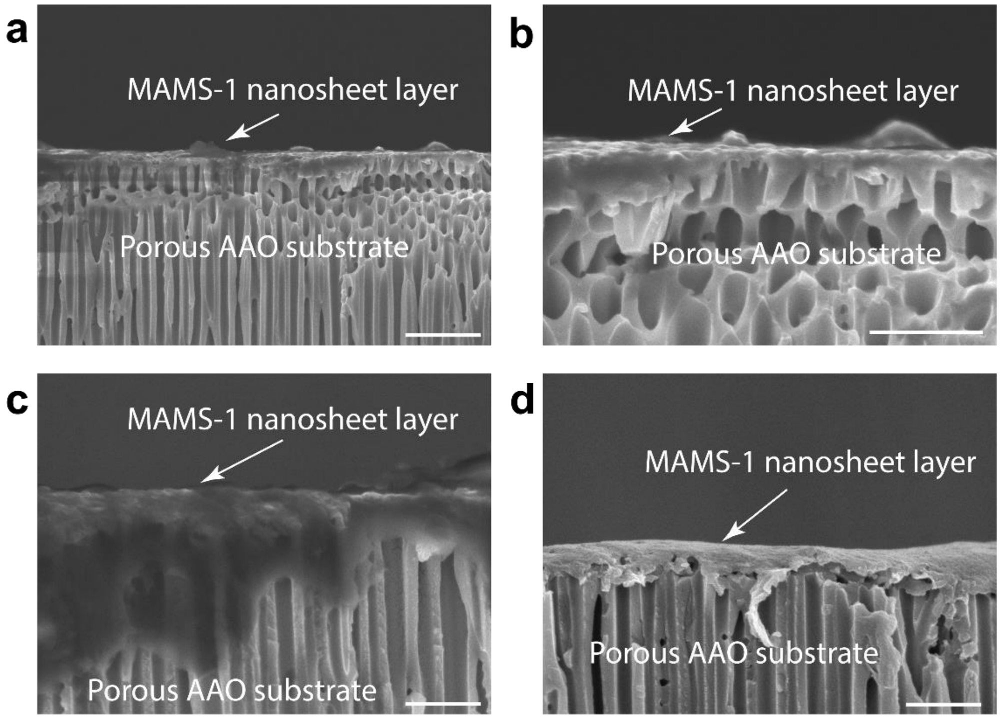

Supplementary Figure 8│SEM images (cross section) of MAMS-1 membranes. **a-b**, 4-nm membrane. **c**, 12-nm membrane. **d**, 40-nm membrane. Scale bars: (a), 2 μm; (b-d), 1 μm.

Supplementary Note 5: It is difficult to accurately determine the membrane thickness from cross sectional SEM images because of the small dimension and flexibility of MAMS-1 nanosheet layer which can fold around the boundary of the fractured substrate. This problem can be solved by FIB-TEM demonstrated in Figure 3d-3f in the main text.

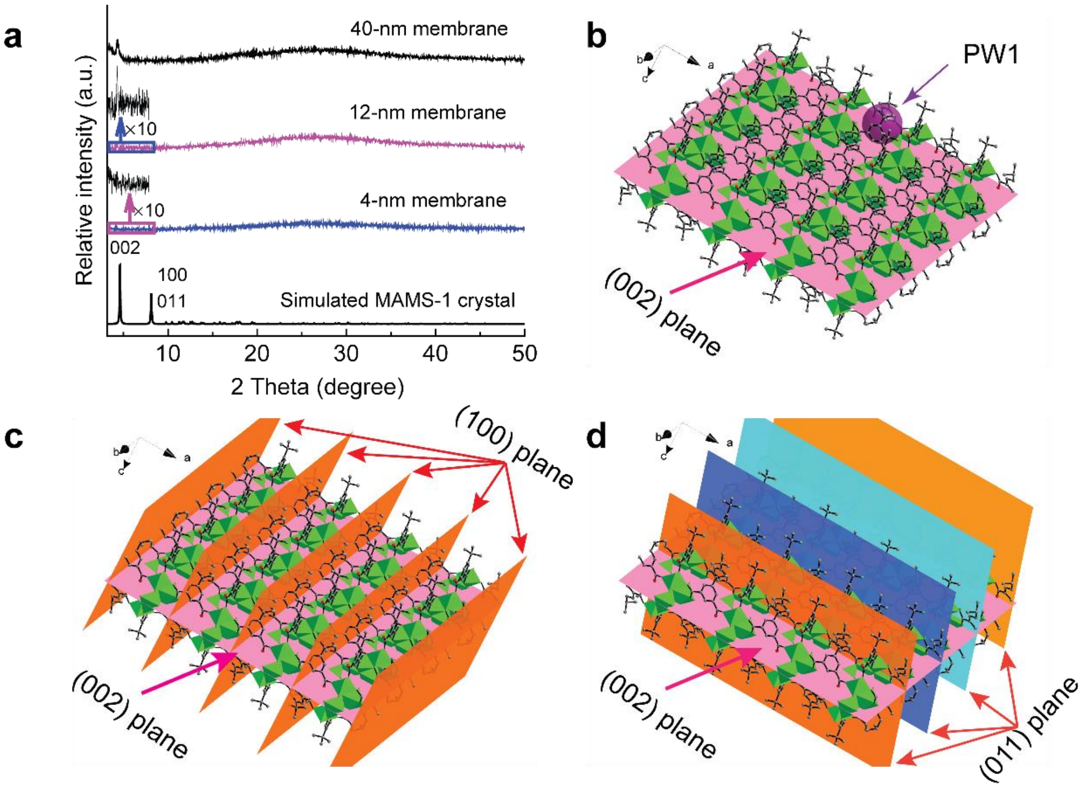

Supplementary Figure 9│**PXRD patterns of MAMS-1 membranes and the corresponding crystal planes.** **a**, PXRD patterns of simulated MAMS-1 crystal, 4-nm membrane, 12-nm membrane, and 40-nm membrane. **b**, The (002) crystal plane in MAMS-1 crystal structure. **c**, The (002) and (100) crystal planes in MAMS-1 crystal structure. **d**, The (002) and (011) crystal planes in MAMS-1 crystal structure.

Supplementary Note 6: The PXRD pattern of simulated MAMS-1 crystal features two peaks at 4.62 and 8.14 ° corresponding to (002), (100) and (011) crystal planes, respectively (Supplementary Figure 9a). The (002) crystal plane is parallel to the basal plane of MAMS-1 nanosheets (Supplementary Figure 9b), while the (100) and (011) crystal planes are almost perpendicular to it (Supplementary Figure 9c and Supplementary Figure 9d). In the case of the 40-nm membrane, only the peak from (002) crystal plane is detectable (Supplementary Figure 9a), indicating the oriented stacking of MAMS-1 nanosheets along the basal plane exposing PW1 (Supplementary Figure 9b) with small aperture suitable for molecular sieving gas separation. On the contrary, no PXRD peak can be detected from the 4-nm membrane and the 12-nm membrane (Supplementary Figure 9a). Considering the identical fabrication procedure between the membranes, the missing PXRD peak of the 4-nm membrane and the 12-nm membrane can be attributed to the ultra-small thickness of the membranes which prevents the effective X-ray scattering for detectable PXRD signals.

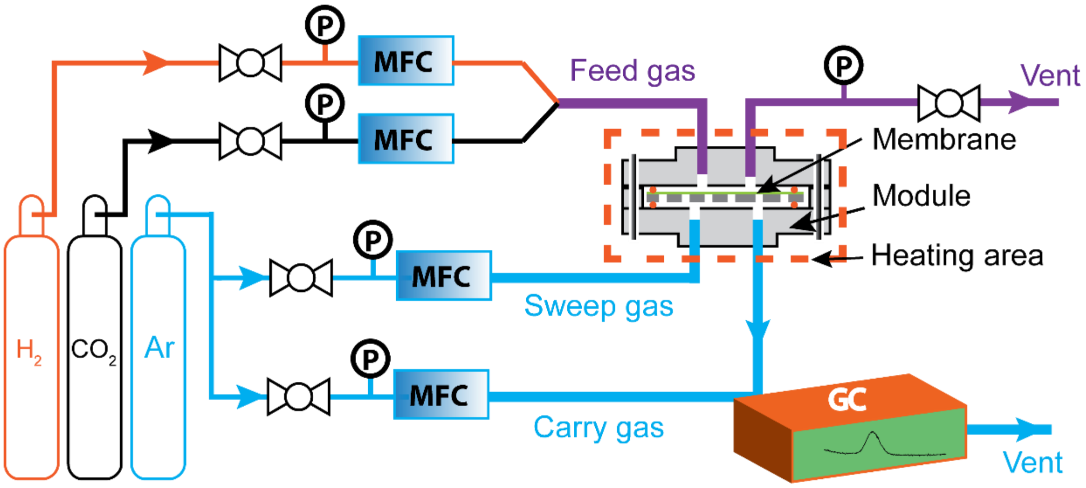

Supplementary Figure 10 | Apparatus scheme for gas permeation tests.

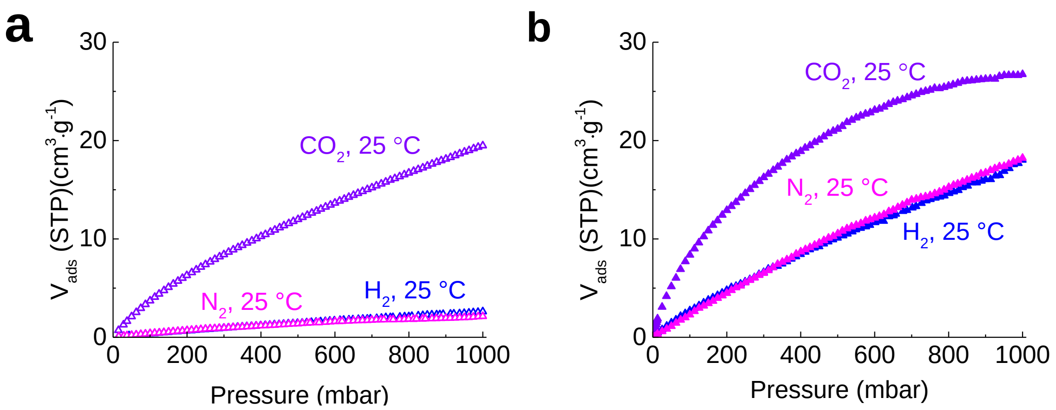

Supplementary Figure 11 | Gas sorption isotherms at 25 ℃. **a**, MAMS-1 crystals. **b**, Exfoliated MAMS-1 nanosheets.

Supplementary Note 7: Both MAMS-1 crystals and exfoliated MAMS-1 nanosheets exhibit preferential adsorption toward CO_2 over H_2 and N_2. Notably, the gas uptakes of MAMS-1 nanosheets are much higher than that of the MAMS-1 crystals, indicating the effective exfoliation which makes the interlayer regions of MAMS-1 crystals accessible to gases in the exfoliated nanosheets.

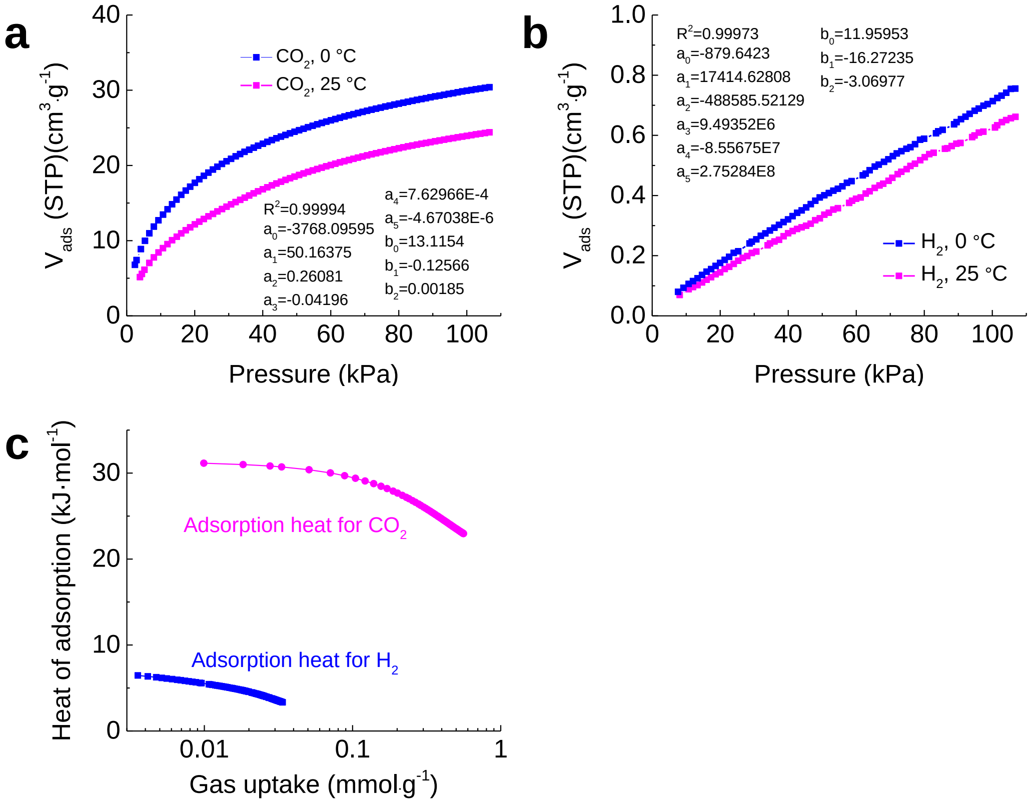

Supplementary Figure 12 | CO_2 and H_2 sorption isotherms and adsorption heats of MAMS-1 crystals. **a**, CO_2 sorption isotherms. **b**, H_2 sorption isotherms. **c**, adsorption heat for H_2 and CO_2.

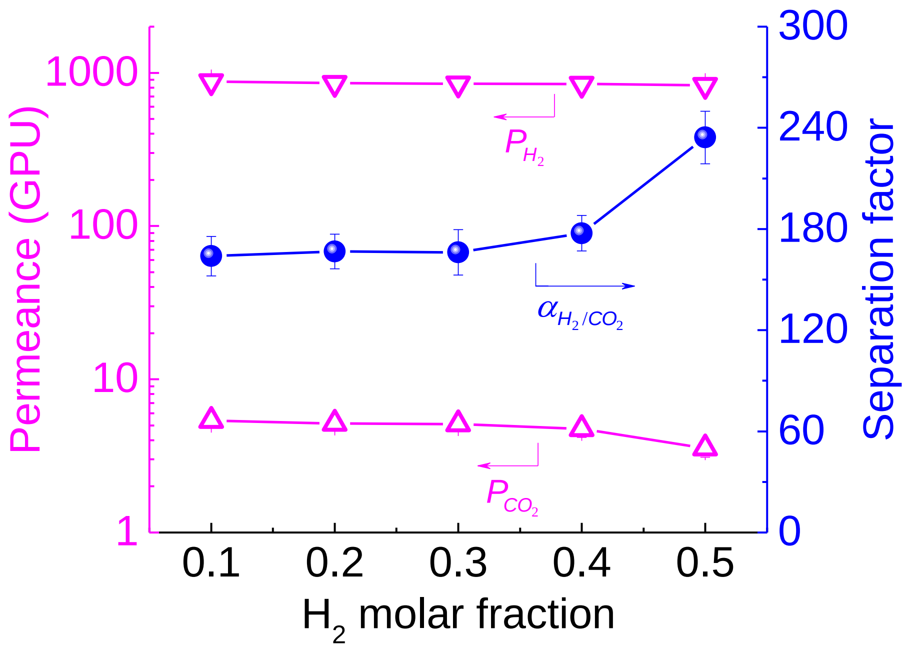

Supplementary Figure 13│The effect of H_2 molar fraction on the separation performance of H_2/CO_2 mixtures using 40-nm membrane.

Supplementary Note 8: The 40-nm membrane demonstrates stable separation performance for H_2/CO_2 mixtures with various H_2 molar fractions ranging from 0.1 to 0.5. The H_2 permeance was hardly affected by the CO_2 molar fraction, while the CO_2 permeance slightly decreased when the H_2 molar fraction increased to higher than 0.4, leading to an increase of the separation factor. This phenomenon might be due to the reduced adsorption of CO_2 molecules at lower CO_2 partial pressures (Supplementary Figure 11 and Supplementary Figure 12).

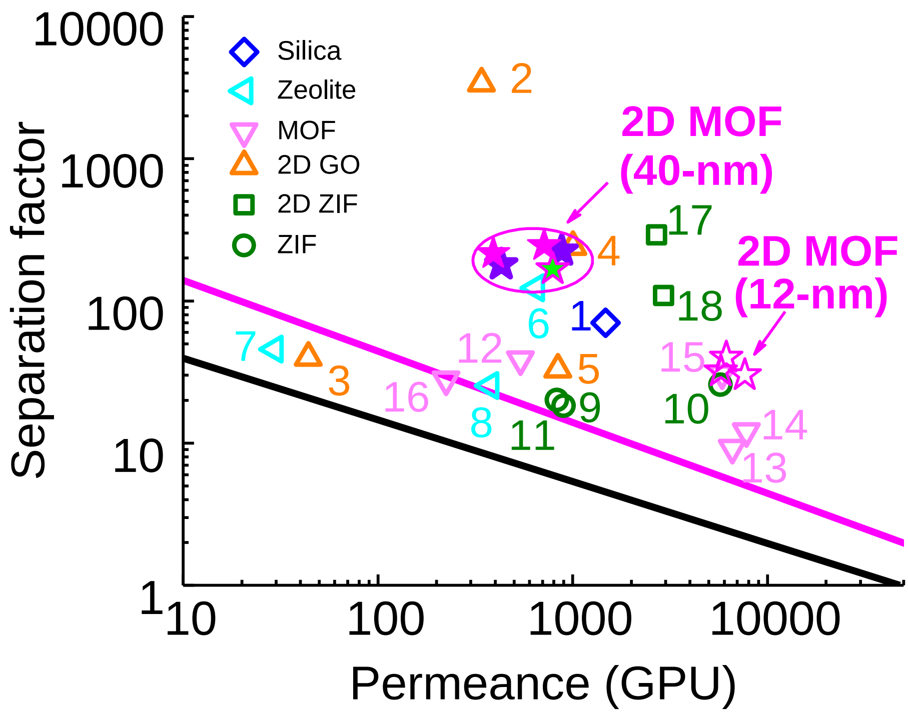

Supplementary Figure 14 | H_2 permeance versus H_2/CO_2 separation factor of 12-nm and 40-nm membranes in this study along with several reference inorganic porous membranes for H_2/CO_2 separation. The magenta line indicates the 2010 upper bound of microporous inorganic membranes for H_2/CO_2 separation[7], and the black line indicates the Robeson 2008 upper bound of pure polymeric membranes for H_2/CO_2 separation[8], assuming a membrane thickness of 100 nm. Please refer to Supplementary Table 3 for detailed test conditions of the presented data points.

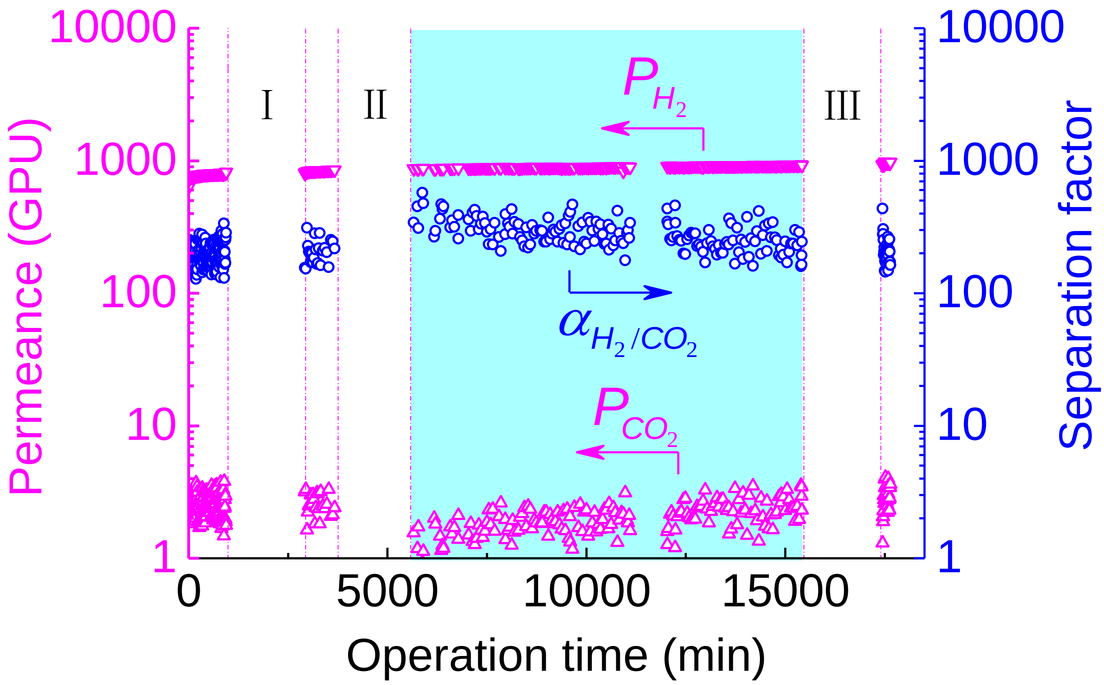

Supplementary Figure 15│Stability evaluation of the 40-nm membrane for the separation of equimolar H_2/CO_2 mixture at 25 ℃.

Supplementary Note 9: Here is the full view of the long-term stability evaluation of the 40-nm membrane for the separation of equimolar H_2/CO_2 mixture at 25 ℃ for more than 300 h in total. The blank periods from left to right represent single gas permeation (He, H_2, CO_2, O_2, N_2, CH_4, and SF_6, period I) and separation of H_2/CO_2 mixtures with different H_2 molar fractions (period II and III), respectively. The highlighted region represents the continuous separation of equimolar H_2/CO_2 mixture for 10,000 min, as shown in Figure 4b.

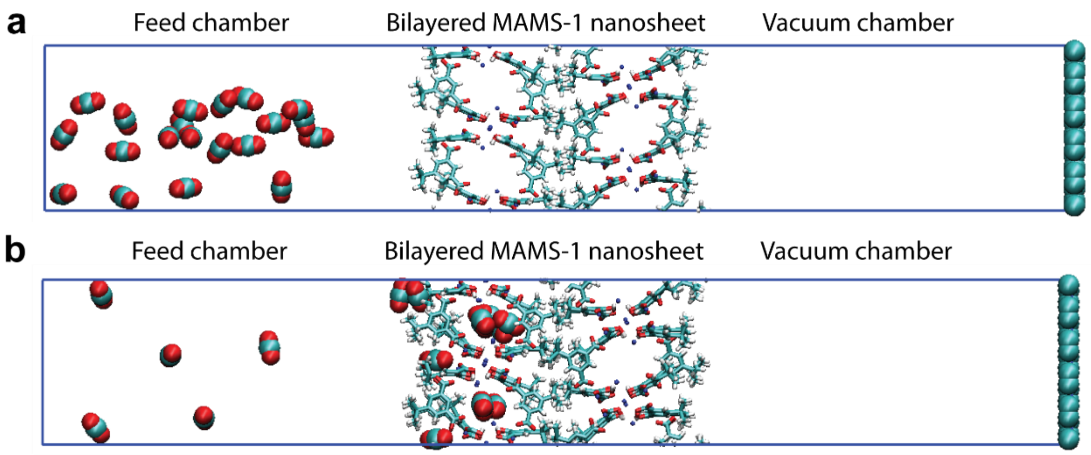

Supplementary Figure 16 | Simulation system for the permeation of pure CO_2 through a bilayered MAMS-1 nanosheet (a) and snapshot after 80 ns of simulation (b). 20 CO_2 molecules and a vacuum are on the left and right of the nanosheet, respectively. A graphene plate is exerted to separate the feed and permeate chambers. Color of the atoms: C, cyan; Ni, blue; O, red; H, white.

Supplementary Note 10: During the 80 ns MD simulation, only 65 % of CO_2 molecules can penetrate into PW2 sandwiched in the first MAMS-1 layer but remain trapped there, and no CO_2 molecule can permeate through the entire bilayered MAMS-1 nanosheet.

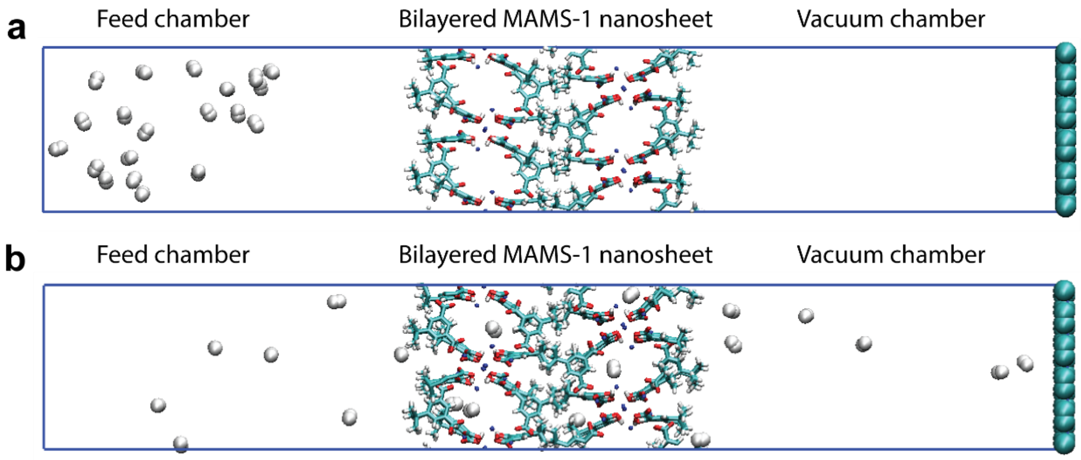

Supplementary Figure 17 | Simulation system for the permeation of pure H_2 through a bilayered MAMS-1 nanosheet (a) and snapshot after 80 ns of simulation (b). 20 H_2 molecules and a vacuum are on the left and right of the nanosheet, respectively. A graphene plate is exerted to separate the feed and permeate chambers. Color of the atoms: C, cyan; Ni, blue; O, red; H, white.

Supplementary Note 11: After the 80 ns MD simulation, 35 % of H_2 molecules can penetrate into PW2 and 35 % of H_2 molecules can permeate through the entire bilayered MAMS-1 nanosheet.

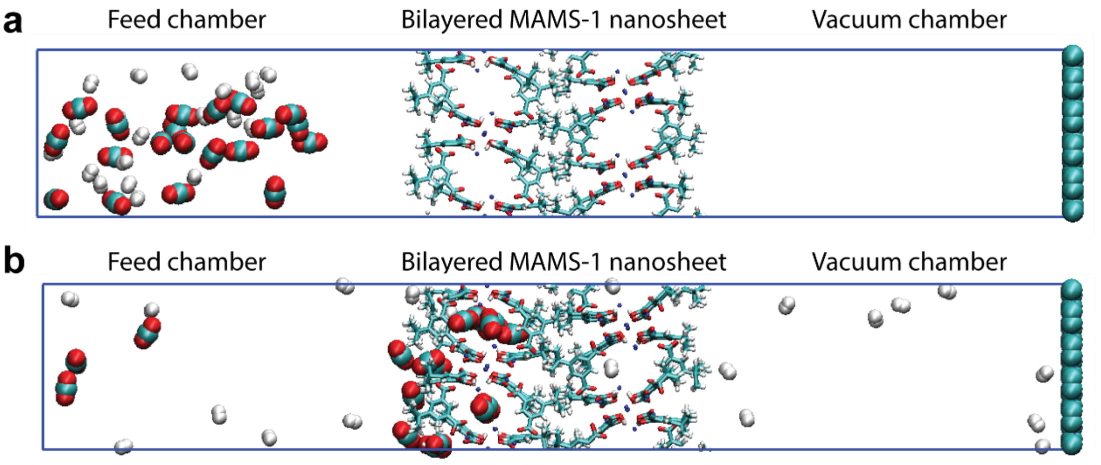

Supplementary Figure 18 | Simulation system for the permeation of an equimolar H_2/CO_2 mixture through a bilayered MAMS-1 nanosheet (a) and snapshot after 80 ns of simulation (b, also shown as Figure 4c in the main text). An equimolar mixture of H_2/CO_2 (40 molecules in total) and a vacuum are on the left and right of the nanosheet, respectively. A graphene plate is exerted to separate the feed and permeate chambers. Color of the atoms: C, cyan; Ni, blue; O, red; H, white.

Supplementary Note 12: In the case of equimolar H_2/CO_2 mixture, 45 % of H_2 molecules can permeate through the entire bilayered MAMS-1 nanosheet, while CO_2 molecules can only penetrate into PW2 sandwiched in the first MAMS-1 layer and remain trapped there throughout the entire 80 ns.

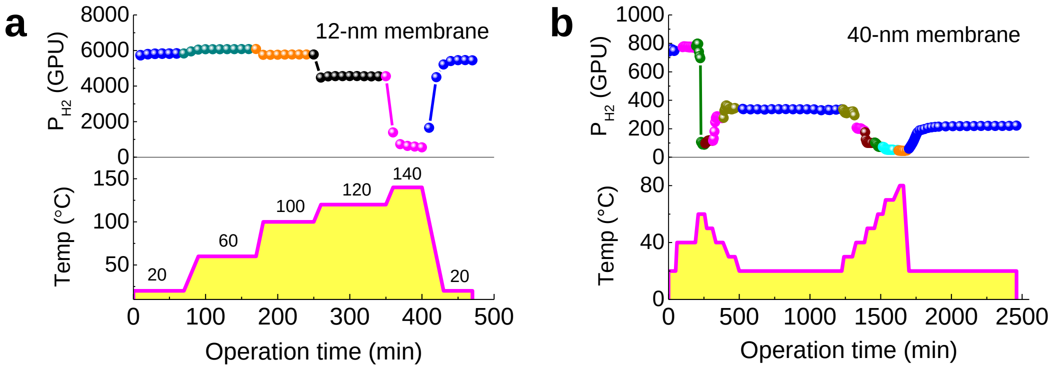

Supplementary Figure 19 | H_2 permeance of 2D MAMS-1 membranes under heating/cooling cycles. a, 12-nm membrane for one cycle. **b**, 40-nm membrane for two cycles.

Supplementary Note 13: The 12-nm membrane also exhibits reversed thermo-switchable feature for H_2 permeation, which is similar to the 40-nm membrane. Notably, after the first heating/cooling cycle, the H_2 permeance of the 12-nm membrane can resume back to the original value. However, the H_2 permeance of the 40-nm membrane can only resume back to half of the original value after first heating/cooling cycle, and keeps decreasing after the second heating/cooling cycle. The detailed reasons remain unknown and further study is needed.

Supplementary Table 1 Specific surface areas determined from nitrogen adsorption isotherm at 77 K.

Sample	S_{BET}[a]/ m^2 g^{-1}	S_{mic}[b]/ m^2 g^{-1}	S_{ext}[c]/ m^2 g^{-1}
MAMS-1 crystals	24.8	11.3	13.5
MAMS-1 nanosheets	126.1	40.5	85.6

[a]: BET surface area.

[b]: Micropore surface area.

[c]: External surface area.

Supplementary Table 2 Gas separation performance for equimolar H_2/CO_2 mixture at room temperature using 2D MAMS-1 membranes.

Membrane	Vol. of DMSO Suspension / mL	P_{H_2} / GPU	Average	α_{H_2/CO_2}	Average
Substrate	0	>20000		1.4	
4-nm	0.5	6670		3	
12-nm	4.5	7640	6516 ±990	30	34 ±5
	4.5	5773		32	
	4.5	6135		40	
40-nm	20	715	553 ±228	245	235 ±14
	20	392		225	

Supplementary Table 3 Detailed test conditions of the data points shown in **Supplementary Figure 14**.

Membrane Type	Symbol	No.	Membrane Material	H_2/CO_2 Ratio	Temp. / ℃	P_{H_2} / GPU	α_{H_2/CO_2}	Ref.
Silica	◇	1	Silica	N.A. [a]	200	1474	70 [b]	9
Graphene Oxide	△	2	GO	50/50	20	341	3400	10
Graphene Oxide	△	3	Thermally treated GO	N.A. [a]	140	44	40 [b]	11
Graphene Oxide	△	4	GO	50/50	25	1002	240	2
Graphene Oxide	△	5	GO	50/50	25	840	33	12
Polycrystalline zeolites	◁	6	Modified MFI	50/50	450	648	123	13
Polycrystalline zeolites	◁	7	Modified MFI	50/50	500	29.5	45.6	14
Polycrystalline zeolites	◁	8	Modified ZSM-5/Silicate	50/50	450	377	25.3	15
Polycrystalline ZIFs	○	9	ZIF-7	50/50	150	899	18.3	16
Polycrystalline ZIFs	○	10	ZIF-95	50/50	325	5747	25.7	17
Polycrystalline ZIFs	○	11	APTES-modified ZIF-90	50/50	225	831	20.1	18
Polycrystalline MOFs	▽	12	JUC-150	50/50	25	539	38.7	19
Polycrystalline MOFs	▽	13	HKUST-1	50/50	25	6595	9.24	20
Polycrystalline MOFs	▽	14	$Zn(BDC)(TED)_{0.5}$	50/50	180	7810	12.1	21
Polycrystalline MOFs	▽	15	NH_2-MIL-53(Al)	50/50	15	224	28	22
Polycrystalline MOFs	▽	16	Amine-modified Mg-MOF-74	50/50	25	5851	30.9	23
2D ZIFs	○	17	$Zn_2(bim)_4$ Nanosheets	50/50	25	2700	291	24
	○	18	$Zn_2(bim)_4$ Nanosheets	20/80	25	2927	109	24
2D MOFs (40-nm)	★	19	MAMS-1 Nanosheets	50/50	20	715	245	This work
2D MOFs (40-nm)	★	20	MAMS-1 Nanosheets	50/50	40	880	225	This work
2D MOFs (40-nm)	★	21	MAMS-1 Nanosheets	20/80	20	790	167	This work
2D MOFs (40-nm)	★	22	MAMS-1 Nanosheets	50/50	20	392	215	This work
2D MOFs (40-nm)	★	23	MAMS-1 Nanosheets	50/50	40	430	194	This work
2D MOFs (12-nm)	☆	24	MAMS-1 Nanosheets	50/50	20	5773-7640	30-40	This work

[a]: Single gas permeation; [b]: Ideal separation factor.

Supplementary References

1. Ogino I, Yokoyama Y, Iwamura S, Mukai SR. Exfoliation of graphite oxide in water without sonication: Bridging length scales from nanosheets to macroscopic materials. *Chem. Mater.* **26**, 3334-3339 (2014).

2. Chi C, *et al.* Facile preparation of graphene oxide membranes for gas separation. *Chem. Mater.* **28**, 2921-2927 (2016).

3. Beckstein O, Biggin PC, Sansom MSP. A hydrophobic gating mechanism for nanopores. *J. Phys. Chem. B* **105**, 12902-12905 (2001).

4. Sun P, *et al.* Selective trans-membrane transport of alkali and alkaline earth cations through graphene oxide membranes based on cation-π interactions. *ACS Nano* **8**, 850-859 (2014).

5. Meyer JC, Geim AK, Katsnelson MI, Novoselov KS, Booth TJ, Roth S. The structure of suspended graphene sheets. *Nature* **446**, 60-63 (2007).

6. Zhu Y, *et al.* Exfoliation of graphite oxide in propylene carbonate and thermal reduction of the resulting graphene oxide platelets. *ACS Nano* **4**, 1227-1233 (2010).

7. Li Y, Liang F, Bux H, Yang W, Caro J. Zeolitic imidazolate framework ZIF-7 based molecular sieve membrane for hydrogen separation. *J. Membr. Sci.* **354**, 48-54 (2010).

8. Robeson LM. The upper bound revisited. *J. Membr. Sci.* **320**, 390-400 (2008).

9. De Vos RM, Verweij H. High-selectivity, high-flux silica membranes for gas separation. *Science* **279**, 1710-1711 (1998).

10. Li H, *et al.* Ultrathin, molecular-sieving graphene oxide membranes for selective hydrogen separation. *Science* **342**, 95-98 (2013).

11. Kim HW, *et al.* Selective gas transport through few-layered graphene and graphene oxide membranes. *Science* **342**, 91-95 (2013).

12. Shen J, Liu G, Huang K, Chu Z, Jin W, Xu N. Subnanometer two-dimensional graphene oxide channels for ultrafast gas sieving. *ACS Nano* **10**, 3398-3409 (2016).

13. Tang Z, Dong J, Nenoff TM. Internal surface modification of MFI-type zeolite membranes for high selectivity and high flux for hydrogen. *Langmuir* **25**, 4848-4852 (2009).

14. Hong Z, Sun F, Chen D, Zhang C, Gu X, Xu N. Improvement of hydrogen-separating performance by on-stream catalytic cracking of silane over hollow fiber MFI zeolite membrane. *Int. J. Hydrogen Energy* **38**, 8409-8414 (2013).

15. Wang H, Lin YS. Synthesis and modification of ZSM-5/silicalite bilayer membrane with improved hydrogen separation performance. *J. Membr. Sci.* **396**, 128-137 (2012).

16. Melgar VMA, Kwon HT, Kim J. Direct spraying approach for synthesis of ZIF-7 membranes by electrospray deposition. *J. Membr. Sci.* **459**, 190-196 (2014).

17. Huang A, Chen Y, Wang N, Hu Z, Jiang J, Caro J. A highly permeable and selective zeolitic imidazolate framework ZIF-95 membrane for H_2/CO_2 separation. *Chem. Commun.* **48**, 10981-10983 (2012).

18. Huang A, Wang N, Kong C, Caro J. Organosilica-functionalized zeolitic imidazolate framework ZIF-90 membrane with high gas-separation performance. *Angew. Chem. Int. Ed.* **51**, 10551-10555 (2012).

19. Kang Z, *et al.* Highly selective sieving of small gas molecules by using an ultra-microporous metal-organic framework membrane. *Energy Environ. Sci.* **7**, 4053-4060 (2014).

20. Ben T, Lu C, Pei C, Xu S, Qiu S. Polymer-supported and free-standing metal-organic framework membrane. *Chem. Eur. J.* **18**, 10250-10253 (2012).

21. Huang A, Chen Y, Liu Q, Wang N, Jiang J, Caro J. Synthesis of highly hydrophobic and permselective metal-organic framework $Zn(BDC)(TED)_{0.5}$ membranes for H_2/CO_2 separation. *J. Membr. Sci.* **454**, 126-132 (2014).

22. Zhang F, *et al.* Hydrogen selective NH_2-MIL-53 (Al) MOF membranes with high permeability. *Adv. Funct. Mater.* **22**, 3583-3590 (2012).

23. Wang N, Mundstock A, Liu Y, Huang A, Caro J. Amine-modified Mg-MOF-74/CPO-27-Mg membrane with enhanced H_2/CO_2 separation. *Chem. Eng. Sci.* **124**, 27-36 (2015).

24. Peng Y, *et al.* Metal-organic framework nanosheets as building blocks for molecular sieving membranes. *Science* **346**, 1356-1359 (2014).
